# Supplementary figures and images for: The intercentriolar fibers function as docking sites of centriolar satellites for cilia assembly
Source: J Cell Biol. 2024 Feb 28;223(4):e202105065. doi: 10.1083/jcb.202105065 (PMC10901237; doi:10.1083/jcb.202105065)

SourceDataF1C

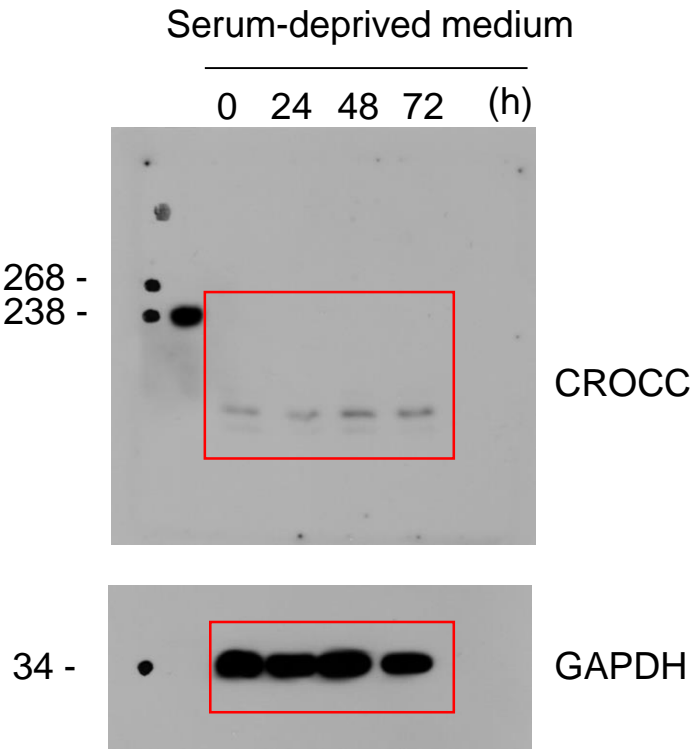

Supplement: SourceData F1 — is the source file for Fig. 1. [file JCB_202105065_SourceDataF1.pdf]

SourceDataF3C

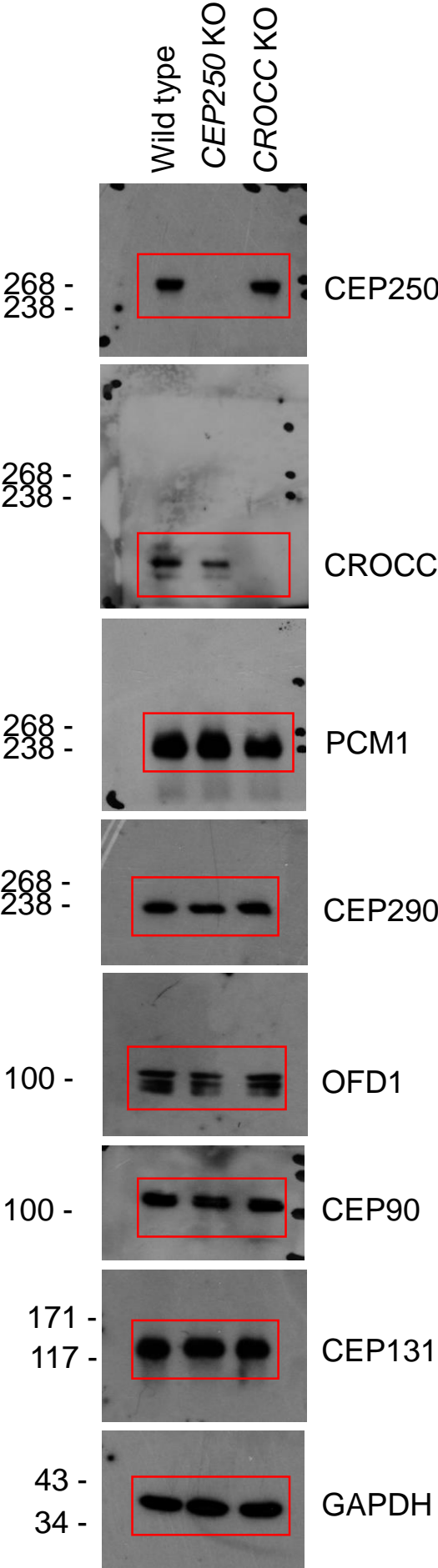

Supplement: SourceData F3 — is the source file for Fig. 3. [file JCB_202105065_SourceDataF3.pdf]

SourceDataF4A

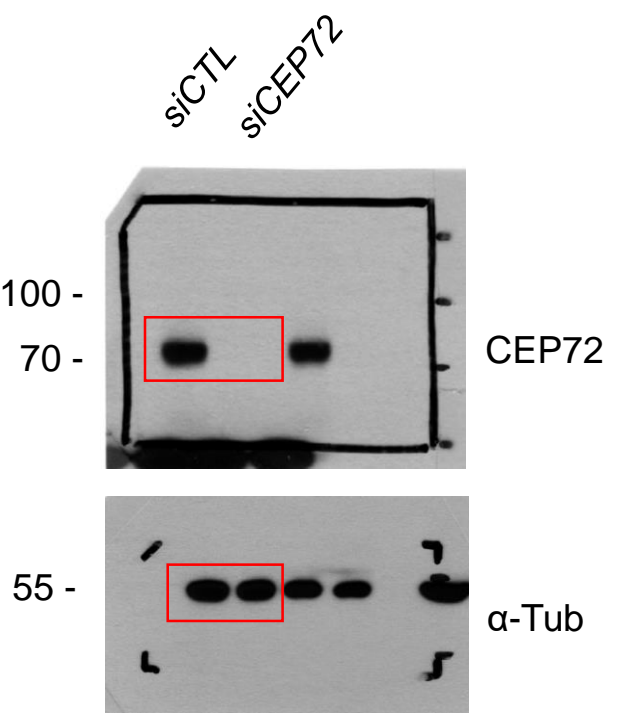

Supplement: SourceData F4 — is the source file for Fig. 4. [file JCB_202105065_SourceDataF4.pdf]

SourceDataF5A

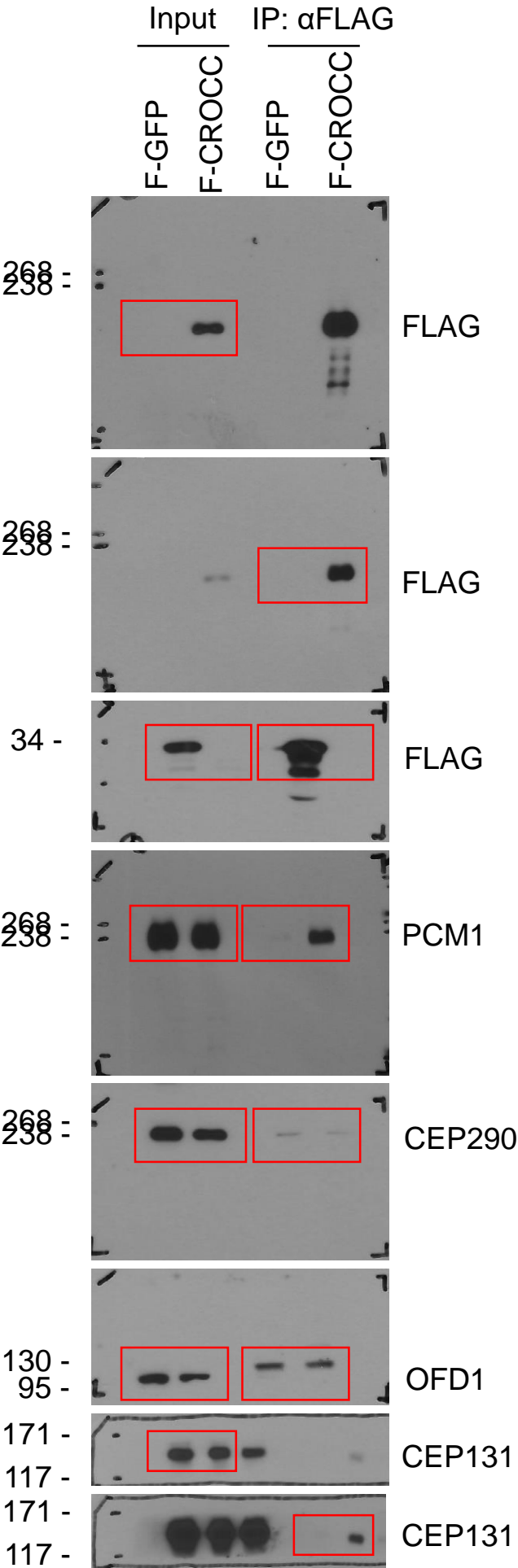

SourceDataF5B

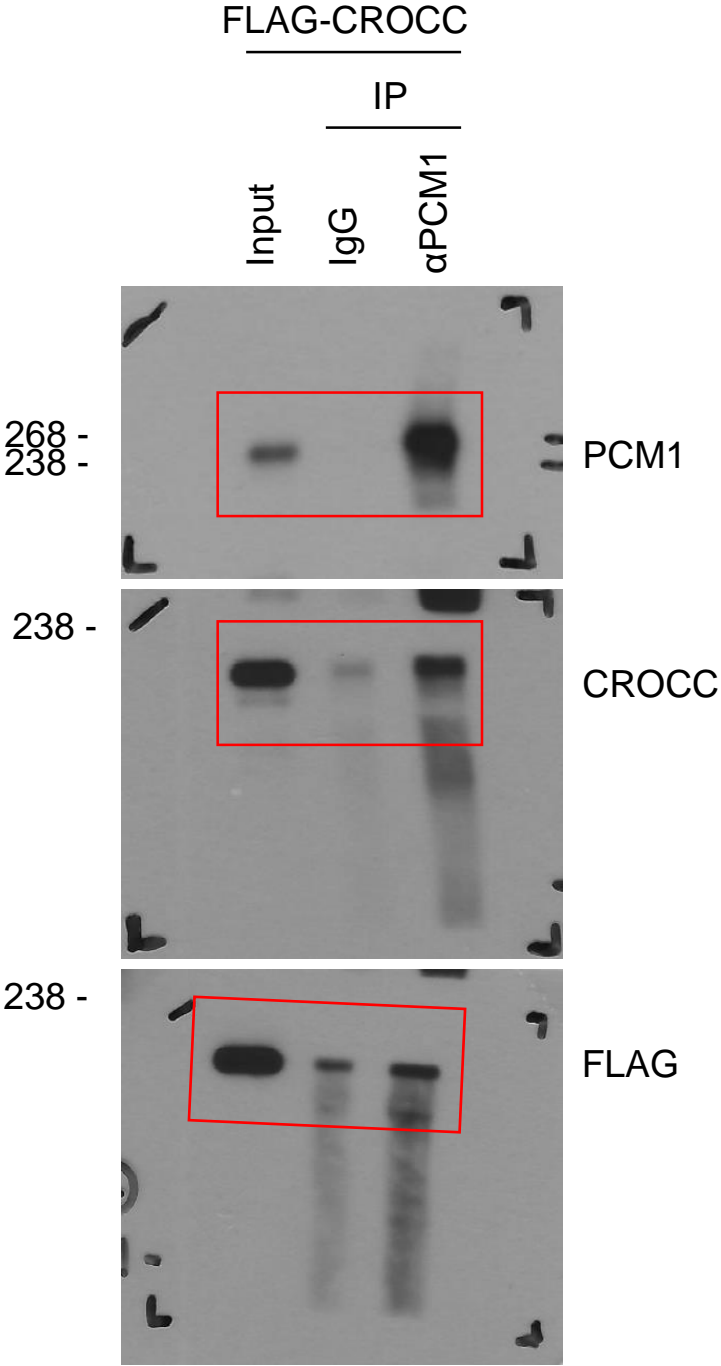

SourceDataF5C

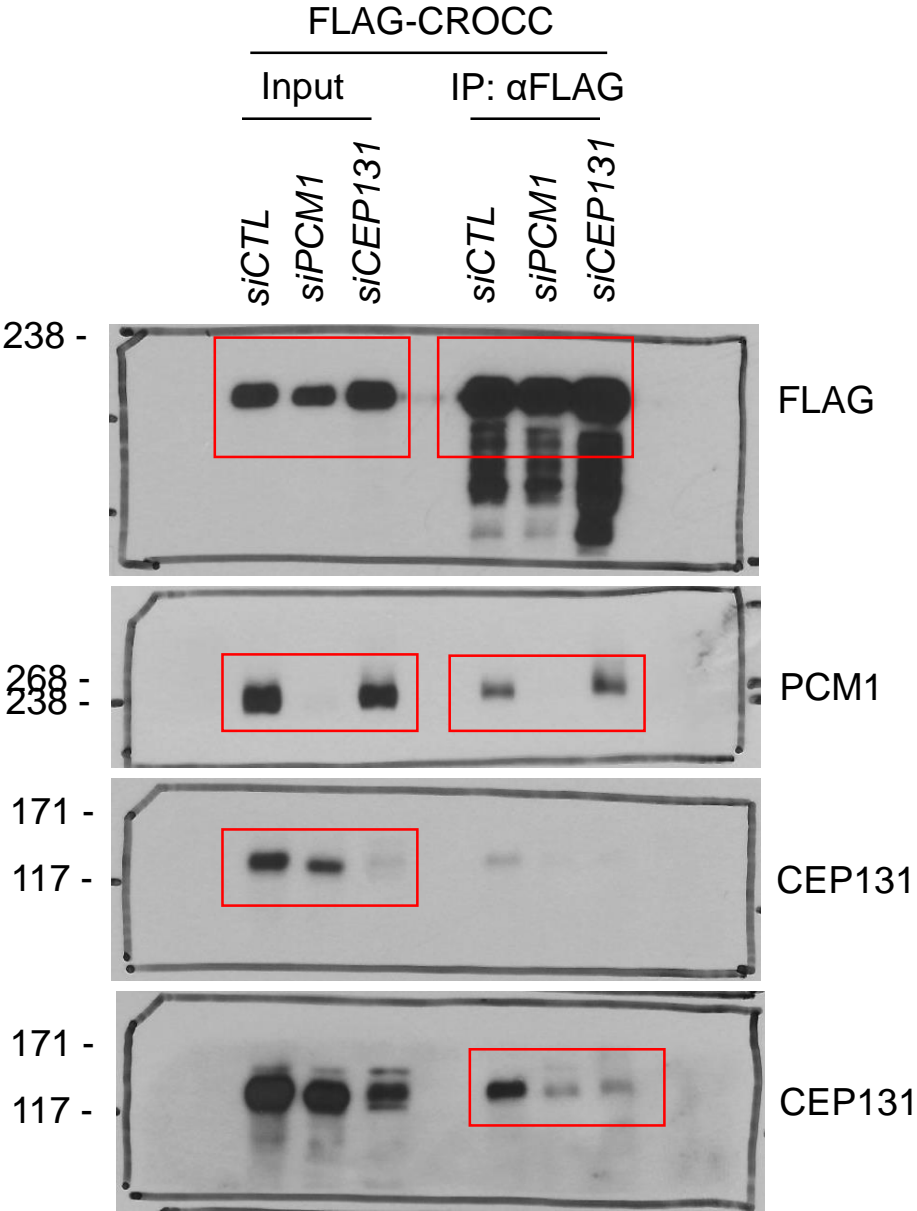

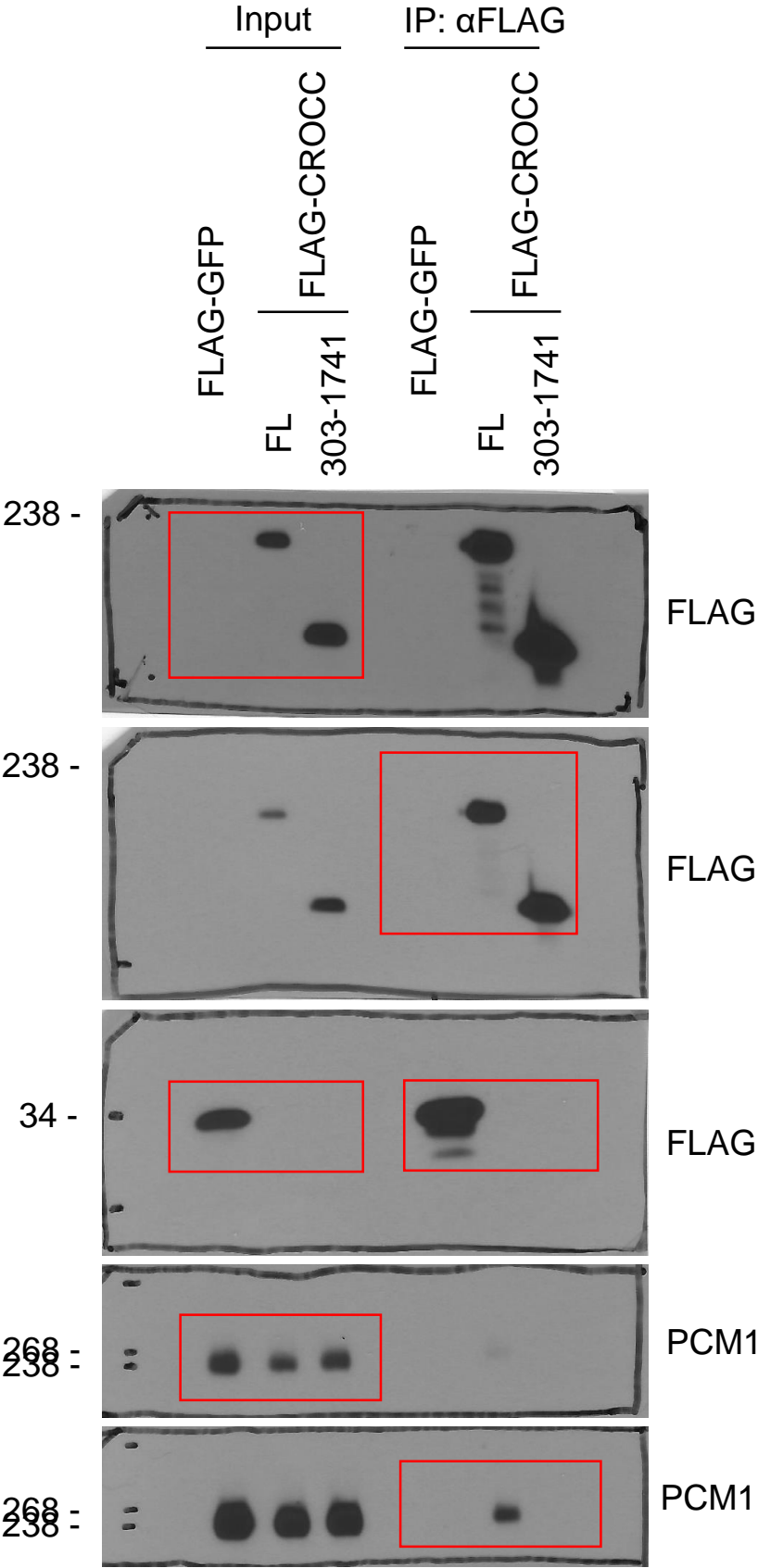

Supplement: SourceData F5 — is the source file for Fig. 5. [file JCB_202105065_SourceDataF5.pdf]

SourceDataF7C

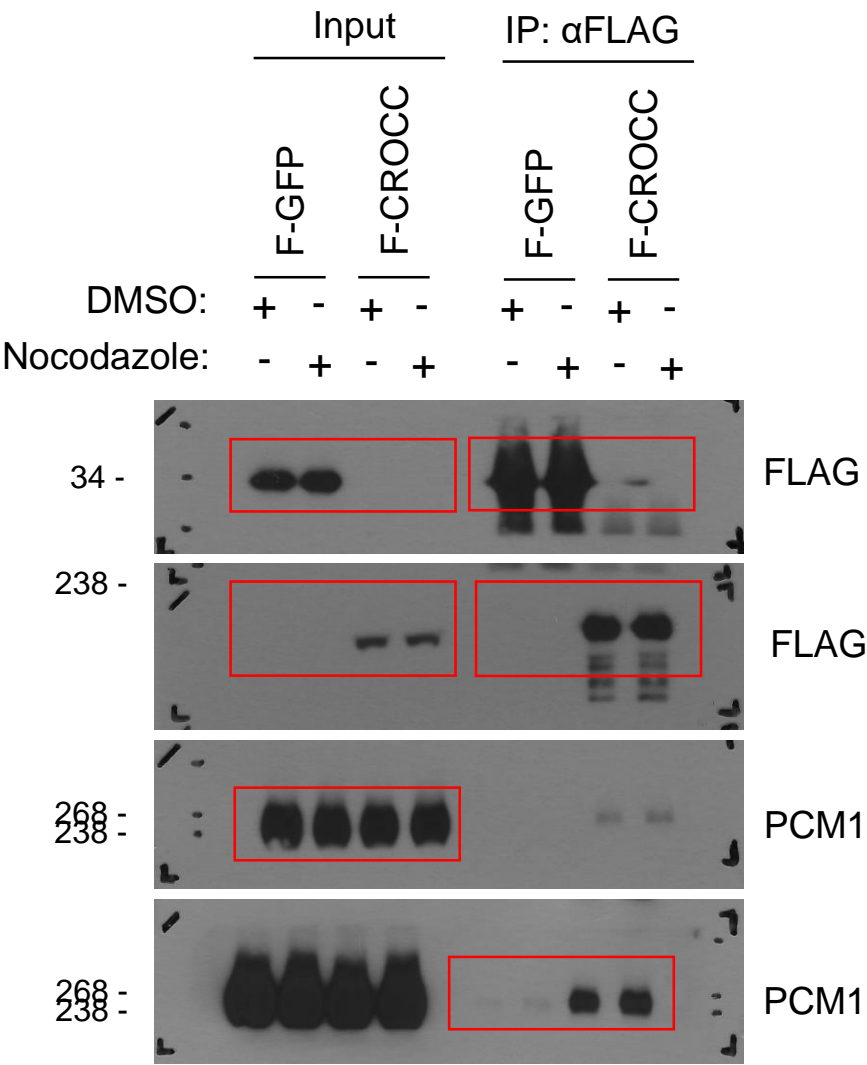

Supplement: SourceData F7 — is the source file for Fig. 7. [file JCB_202105065_SourceDataF7.pdf]

SourceDataF9A

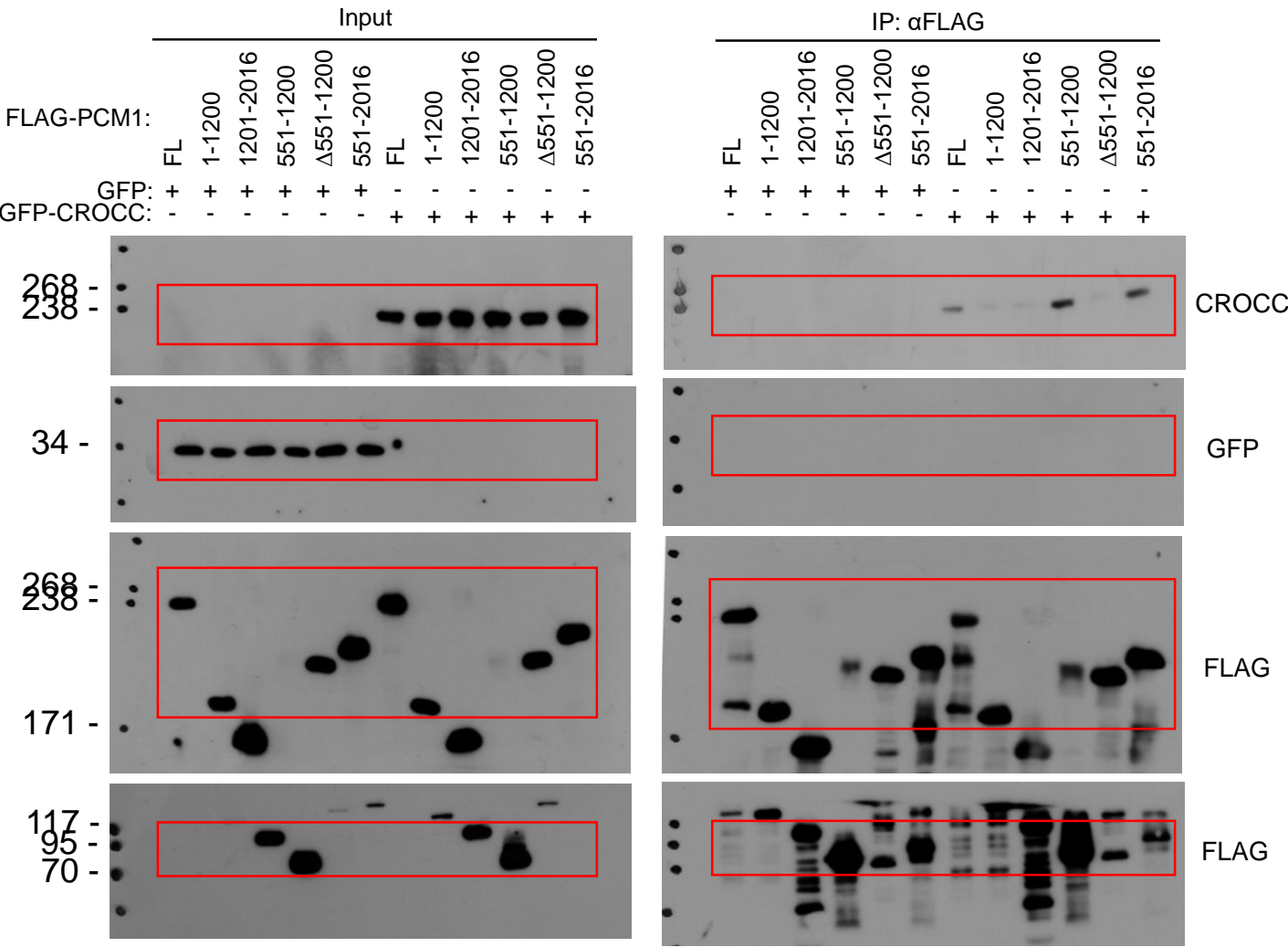

SourceDataF9B

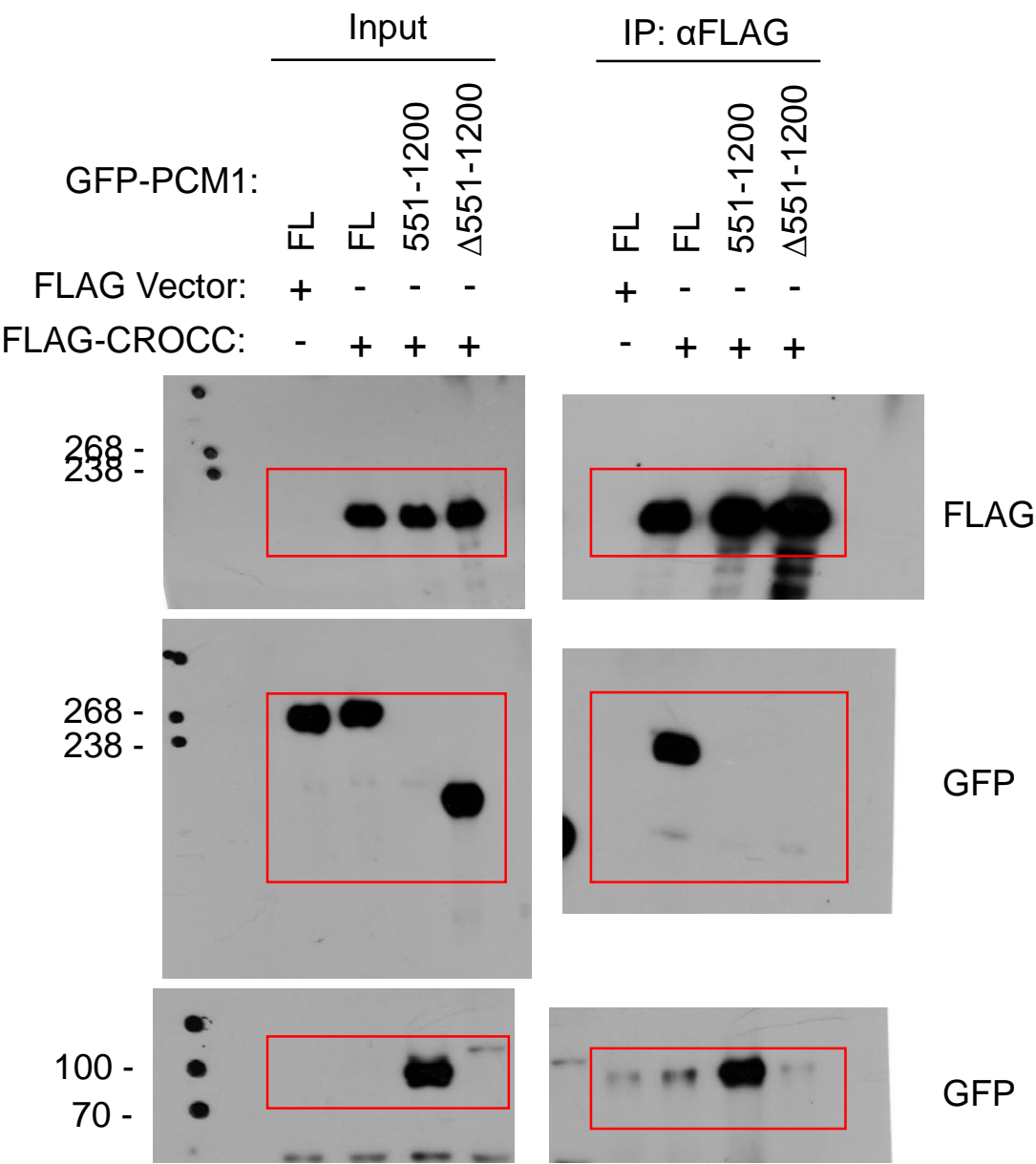

Supplement: SourceData F9 — is the source file for Fig. 9. [file JCB_202105065_SourceDataF9.pdf]

SourceDataSF1B

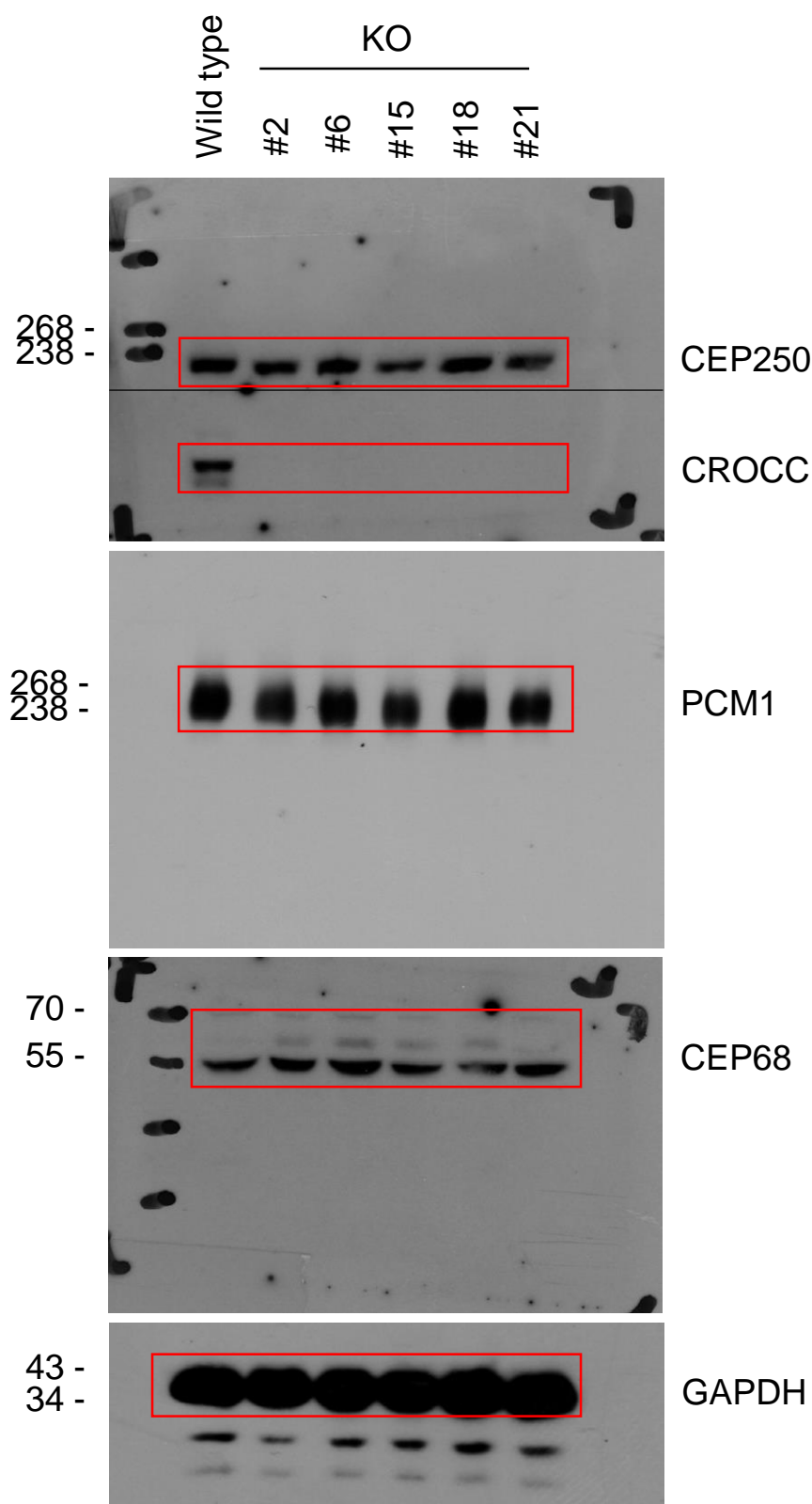

Supplement: SourceData FS1 — is the source file for Fig. S1. [file JCB_202105065_SourceDataFS1.pdf]

SourceDataSF2B

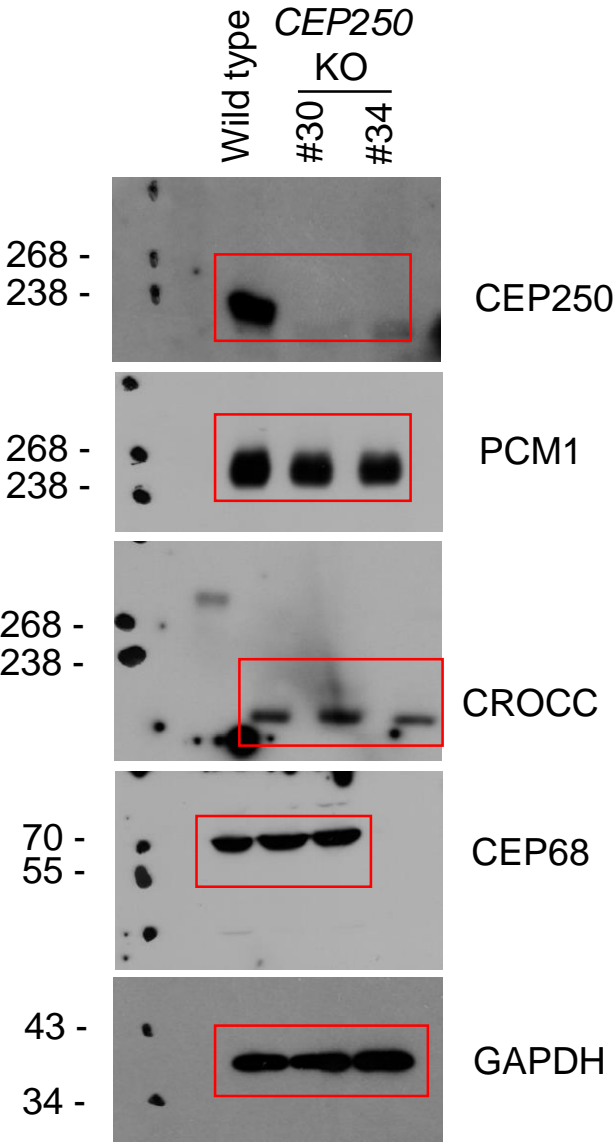

Supplement: SourceData FS2 — is the source file for Fig. S2. [file JCB_202105065_SourceDataFS2.pdf]

SourceDataSF4A

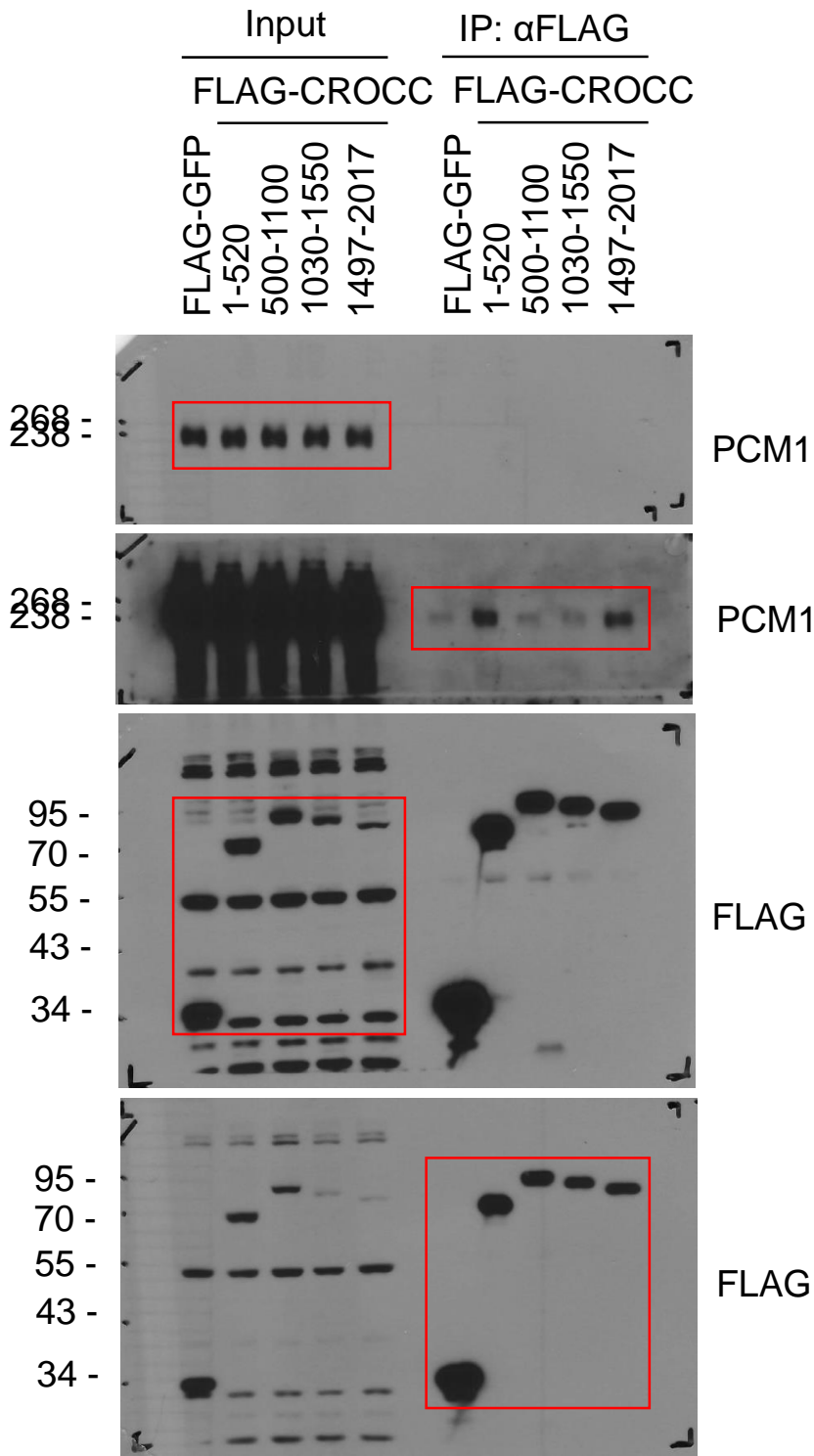

SourceDataSF4B

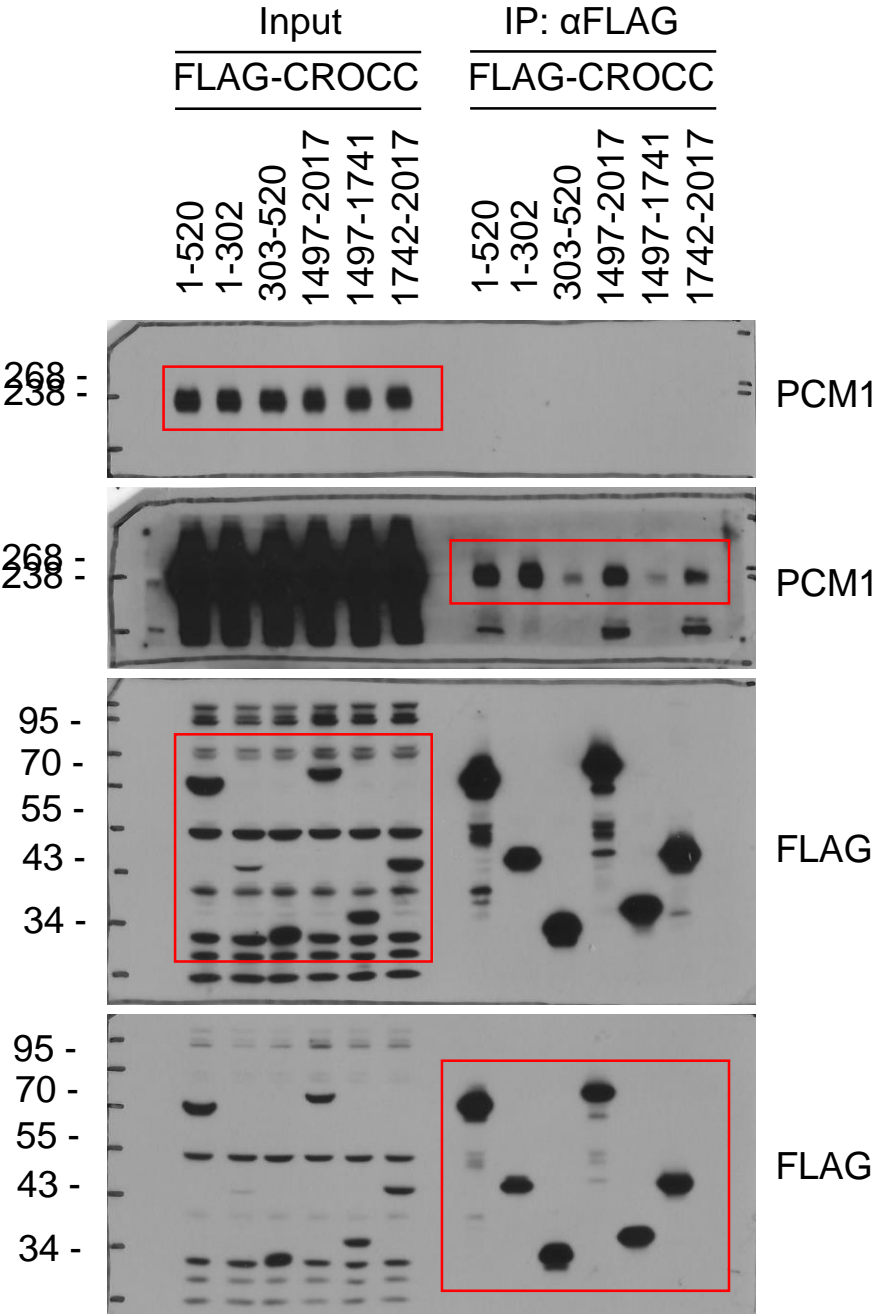

SourceDataSF4C

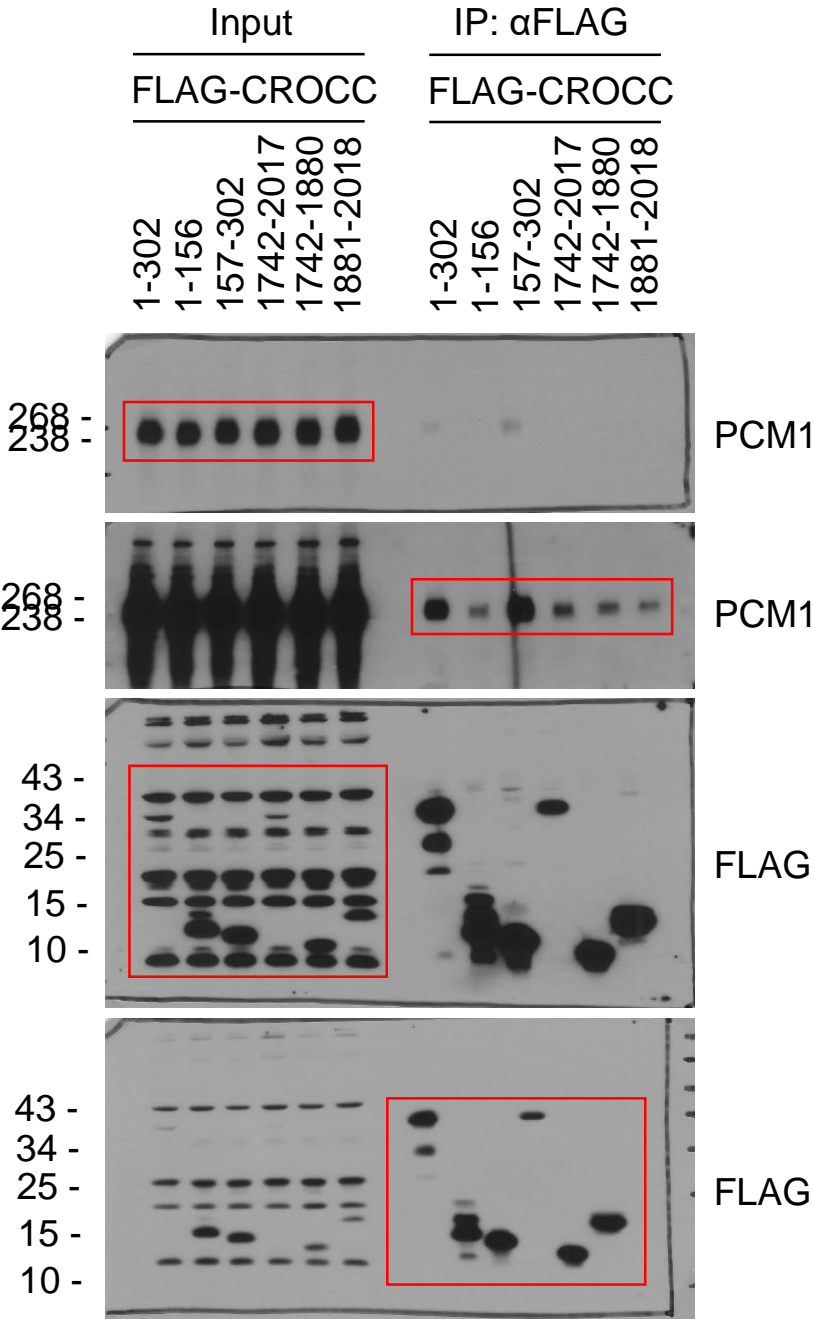

SourceDataSF4D

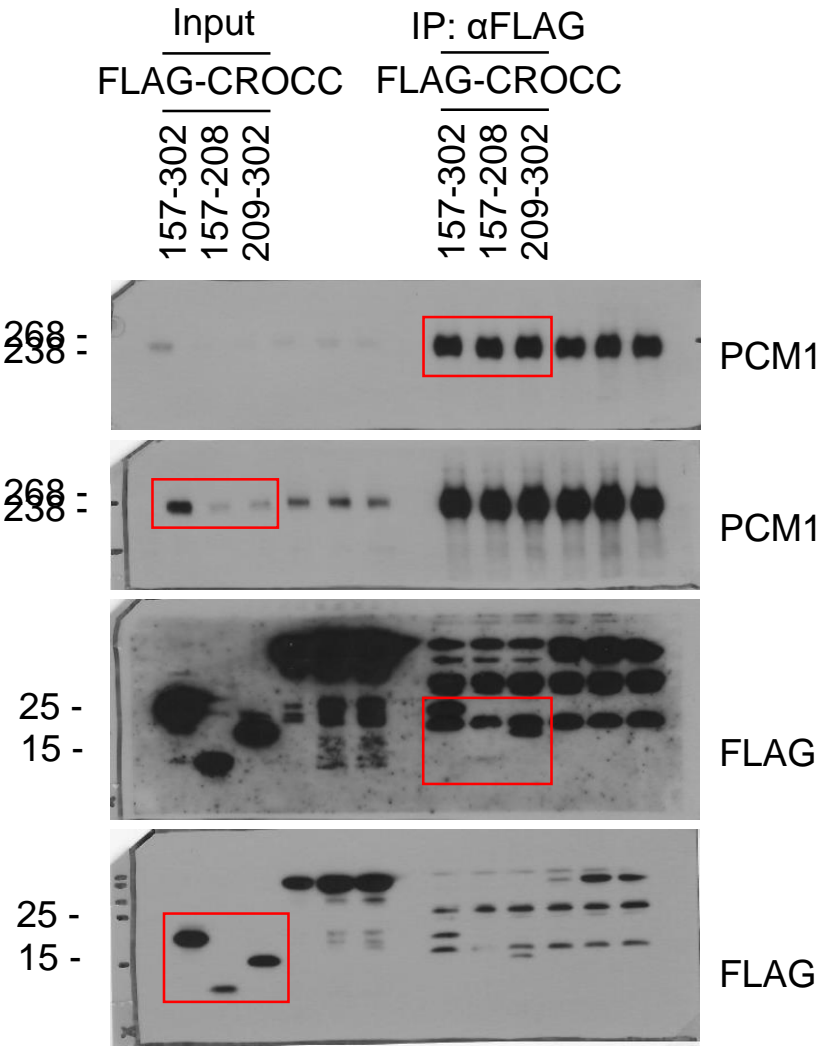

Supplement: SourceData FS4 — is the source file for Fig. S4. [file JCB_202105065_SourceDataFS4.pdf]

SourceDataSF5B

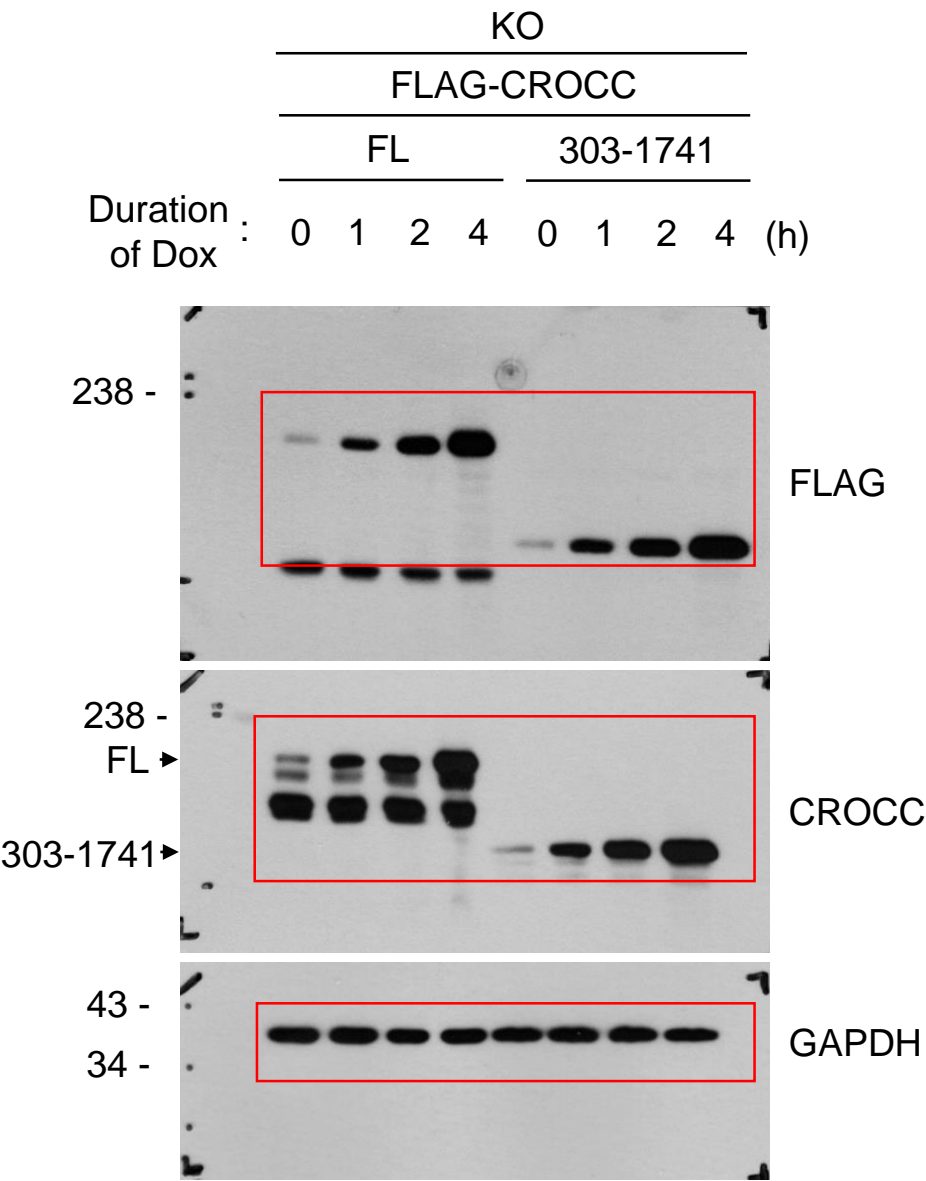

Supplement: SourceData FS5 — is the source file for Fig. S5. [file JCB_202105065_SourceDataFS5.pdf]

SourceDataSF6B

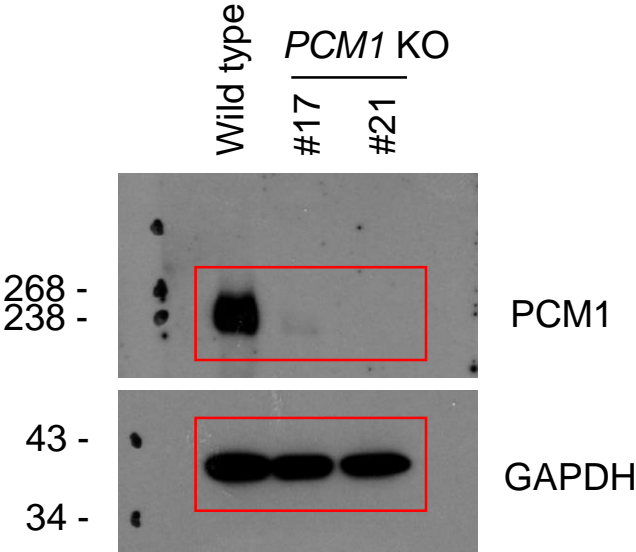

SourceDataSF6C

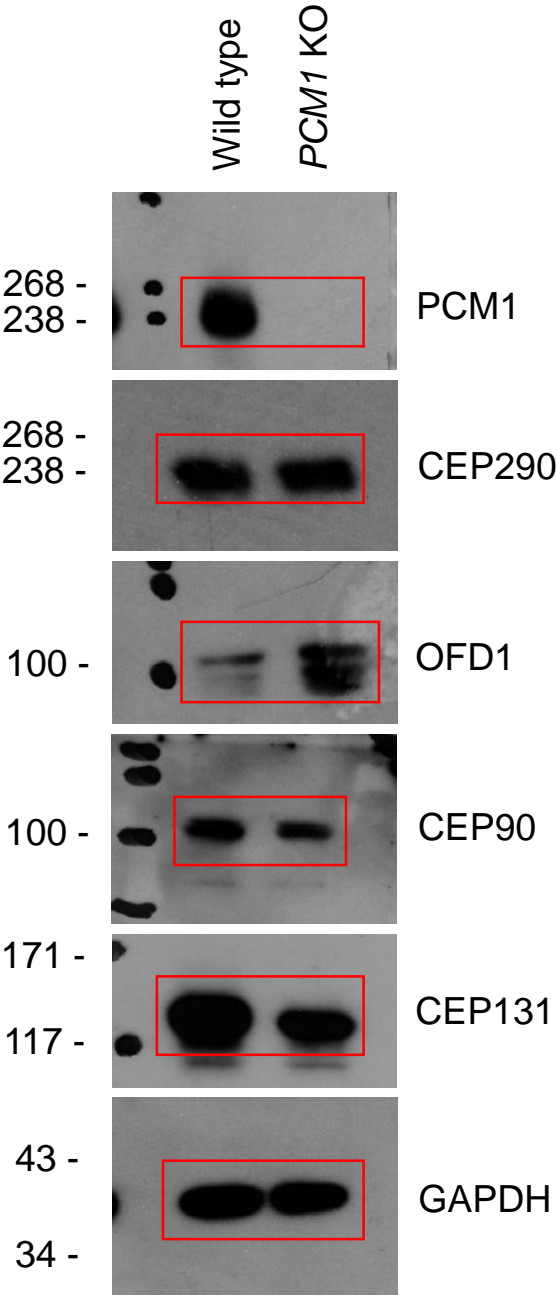

SourceDataSF6H

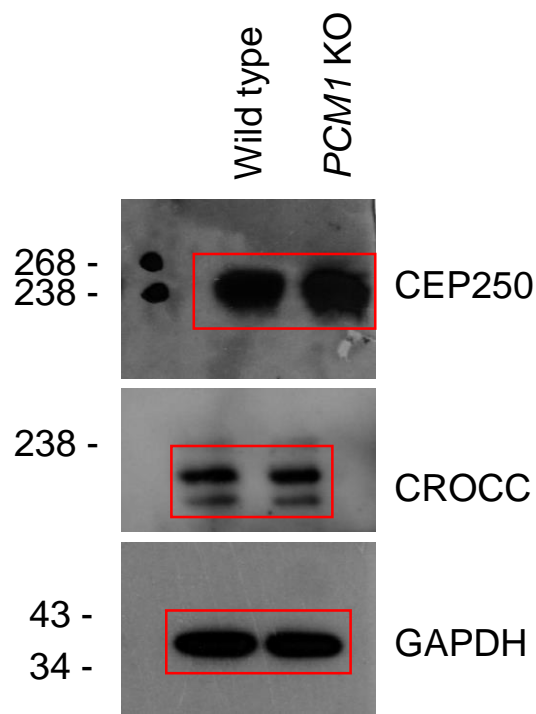

Supplement: SourceData FS6 — is the source file for Fig. S6. [file JCB_202105065_SourceDataFS6.pdf]
