# Supplementary material for: The intercentriolar fibers function as docking sites of centriolar satellites for cilia assembly
Source: J Cell Biol. 2024 Feb 28;223(4):e202105065. doi: 10.1083/jcb.202105065 (PMC10901237; doi:10.1083/jcb.202105065)

SourceDataF8D

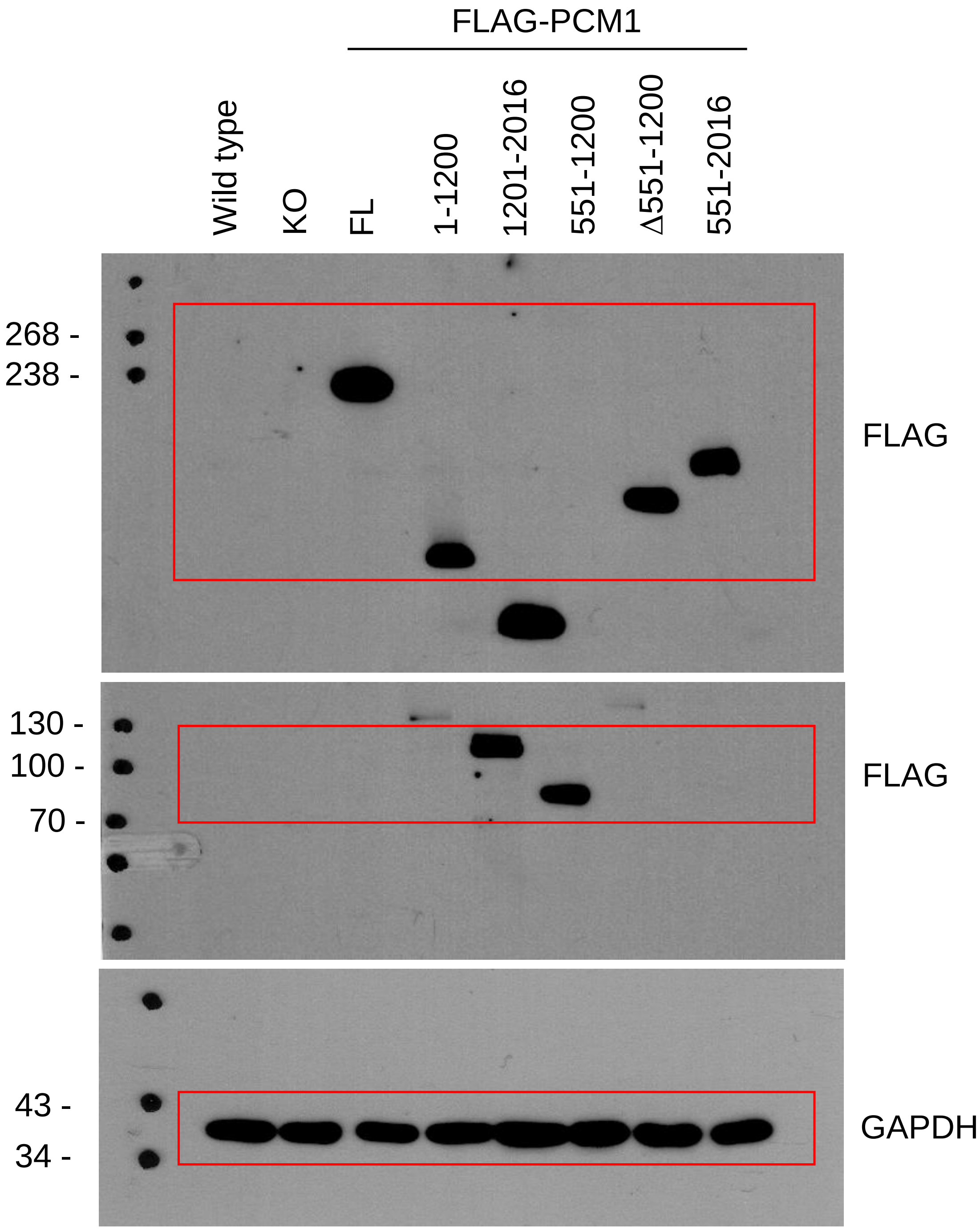

Supplement: SourceData F8 — is the source file for Fig. 8. [file JCB_202105065_SourceDataF8.pdf]
